# Use of patient flow analysis to improve patient visit efficiency by decreasing wait time in a primary care-based disease management programs for anticoagulation and chronic pain: a quality improvement study

**DOI:** 10.1186/1472-6963-7-8

**Published:** 2007-01-15

**Authors:** Nicholas M Potisek, Robb M Malone, Betsy Bryant Shilliday, Timothy J Ives, Paul R Chelminski, Darren A DeWalt, Michael P Pignone

**Affiliations:** 1 Division of General Internal Medicine and Clinical Epidemiology, Department of Medicine, School of Medicine, University of North Carolina at Chapel Hill, Chapel Hill, North Carolina, USA; 2 Division of Pharmacotherapy and Experimental Therapeutics, School of Pharmacy, University of North Carolina at Chapel Hill, Chapel Hill, North Carolina, USA; 3 Center for Excellence in Chronic Illness Care, UNC Health System, Chapel Hill, NC, Chapel Hill, North Carolina, USA

## Abstract

**Background:**

Patients with chronic conditions require frequent care visits. Problems can arise during several parts of the patient visit that decrease efficiency, making it difficult to effectively care for high volumes of patients. The purpose of the study is to test a method to improve patient visit efficiency.

**Methods:**

We used Patient Flow Analysis to identify inefficiencies in the patient visit, suggest areas for improvement, and test the effectiveness of clinic interventions.

**Results:**

At baseline, the mean visit time for 93 anticoagulation clinic patient visits was 84 minutes (+/- 50 minutes) and the mean visit time for 25 chronic pain clinic patient visits was 65 minutes (+/- 21 minutes). Based on these data, we identified specific areas of inefficiency and developed interventions to decrease the mean time of the patient visit. After interventions, follow-up data found the mean visit time was reduced to 59 minutes (+/-25 minutes) for the anticoagulation clinic, a time decrease of 25 minutes (t-test 39%; p < 0.001). Mean visit time for the chronic pain clinic was reduced to 43 minutes (+/- 14 minutes) a time decrease of 22 minutes (t-test 34 %; p < 0.001).

**Conclusion:**

Patient Flow Analysis is an effective technique to identify inefficiencies in the patient visit and efficiently collect patient flow data. Once inefficiencies are identified they can be improved through brief interventions.

## Background

Optimal ambulatory care for patients with chronic conditions requires redesigning the traditional office practice that developed to meet the demands of acute illnesses [1]. Additionally, office practices must efficiently care for high volumes of patients to remain financially viable. Patients with serious and/or multiple chronic health conditions require complex care and make it more difficult to manage high patient volumes. Unorganized patient flow processes increase waiting times and decrease patient satisfaction [2]. Problems can arise at any one of several parts of the office visit, from check-in, to initial nurse intake, to the provider visit, and through check-out.

In one study conducted in 1,789 ambulatory care facilities nationwide, patient satisfaction with outpatient care was influenced strongly by the amount of time the patient spent waiting for care [3]. It was suggested that facilities in teaching organizations and medical schools tend to have longer waits then non-teaching facilities. Another study conducted in an urgent care department of a large land-grant university for medical care evaluated 323 patients found the most important waiting time was the total time patients spent waiting to see the clinician [4]. Decreases in patient satisfaction can affect patient return rates, a necessary component of treating patients with chronic conditions. Quality improvement efforts can help to overcome the barriers to effective patient flow by decreasing patient waiting time, thus improving the efficiency of care.

The Patient Flow Analysis (PFA) process outlines the care process, and measures time spent in each phase of the clinic visit, a potentially effective and efficient technique to collect data and evaluate the effect of interventions to improve patient visit efficiency by decreasing wait time in clinic. Once PFA is performed in clinic, potential targets for improvement can be identified to reduce bottleneck effects in the patient visit, and provide objective data to improve utilization of existing resources. If measures are not easily obtainable, data collection can be an impediment to successful change. Easily replicated in clinic settings, PFA allows staff to evaluate their services, identify problems, and attempt to develop workable solutions fostering a sense of ownership of both problems and solutions among clinic staff. In an early evaluation of its use in two busy family planning clinics in Kenya, feedback on waiting times at different stages of an office visit were used to re-engineer work flow, resulting in 33 to 50% reductions in total visit length [5]. The Centers for Disease Control has produced freely available software to guide and interpret PFA [6]. In this study, PFA was used to determine if the efficiency of care could be improved by decreasing patient wait time for two groups of patients within an academic internal medicine practice disease management program: those requiring chronic anticoagulation, or receiving treatment for chronic pain.

## Methods

PFA was conducted in two programs, an anticoagulation and a chronic pain management program, which are fully integrated within the UNC General Internal Medicine (UNC-GIM) practice. The UNC-GIM practice cares for over 12,000 patients, with almost 30,000 visits per year. The patient population is approximately 40% African American and 60% white. The practice serves patients with a wide range of socioeconomic backgrounds, including approximately 15% uninsured patients.

For the past six years, the UNC-GIM practice has developed a structured care program for anticoagulation patients, who are managed by a multi-disciplinary, collaborative team consisting of the patient's primary care physician, a clinical pharmacist practitioner, and nurses. The anticoagulation program currently has 287 active patients, 58% of the patients are male, the average age is 61 years old, and 65% Caucasian. The majority (42%) of the patients suffer from arterial fibrillation, 35% have prophylaxis from a venous thromboembolism, and 12% treatment of venous thromboembolism. The anticoagulation clinical pharmacist practitioner is responsible for titrating and monitoring warfarin therapy in a systematic method according to standing protocols.

Similarly, for the past five years, the UNC-GIM practice has used a structured care program for patients with chronic pain, anchored by a clinical pharmacist practitioner. A total of 511 patients have been enrolled, with 186 active patients. Of these patients, 62% of the patients are female, 72% Caucasian, with an average age of 56 years. The majority (61%) suffer from back pain, 40% have neuropathy, and 28% osteoarthritis.

### Patient flow analysis

**Figure 1:**
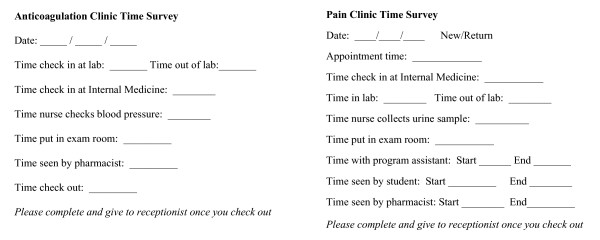
Time-Motion Surveys.

A simple written survey was used to assess the time usage of each patient during anticoagulation or chronic pain visits. The surveys divided the patient visit for each program into several phases, as demonstrated in Figure 1. Clinic personnel, including the front desk staff, nurses, care assistant, and clinical pharmacist practitioners, recorded the time for initiation and completion at each phase during the visit for a two-week period in the anticoagulation clinic and a one-week period in the chronic pain clinic. Initial baseline data for each program were obtained, followed by a review of the mean duration time for each phase and mean visit time. Once key clinic members, including nurses, care assistants, and clinical pharmacist practitioners, identified areas of inefficient patient flow utilizing PFA, interventions were proposed and implemented into each program's patient visit. Baseline data and proposed interventions were reviewed with all clinic staff to ensure a smooth transition of the interventions into the clinic routine.

### Interventions to improve anticoagulation visit efficiency

Interventions to improve patient visit time usage in the anticoagulation clinic involved relocating point-of-care (POC) International Normalized Ratio (INR) testing and increasing nursing support. Originally, the laboratory for POC INR testing for the anticoagulation clinic was in the same building, but on a separate floor. The first intervention involved renovating a patient room located in the same area as the anticoagulation clinical pharmacist practitioner to serve as the POC INR laboratory. The new location increased the proximity between the POC INR laboratory and the anticoagulation visit rooms, therefore simplifying the check-in process. Patients no longer had to check in at two different areas for their POC INR testing and anticoagulation clinic visit. The second intervention involved increasing nursing support by transferring a full-time certified nursing assistant to the clinic staff, which previously consisted of one full-time registered nurse and one full-time licensed practical nurse, for collection of patient vitals and placement of patients into examination rooms. The nursing support prepares patients for assessment by several clinic providers including, anticoagulation, chronic pain, and diabetes clinical pharmacist practitioners, and three to five residents and/or attending physicians. Increasing clinic nursing support helped to place patients into an examination room in a timelier manner after clinic check-in, thereby decreasing the patient load for each nurse.

### Interventions to improve chronic pain visit efficiency

To improve patient visit time usage in the chronic pain clinic, interventions included increasing the clinic responsibilities of the care assistant and making a change in the flow of the patient visit. To decrease the wait time between care assistant and the chronic pain clinical pharmacist practitioner encounters, the care assistant began reviewing and prioritizing chronic pain clinic visits before each clinic session to determine which patients the clinical pharmacist practitioner should assess first, with the care assistant obtaining required information after the assessment. Prior to this trial, patients were seen first by the care assistant and then by the clinical pharmacist practitioner for pain assessment. To decrease redundancy of information collected (e.g., initial pain assessments, including pain generators and most recent dose of opiate medications) and to get patients seen sooner, the care assistant began helping nurses complete initial pain assessments and placing patients into examination rooms.

Change in patient flow during visits involved having nurses collect urine specimens for toxicological screening (UTS) from patients after evaluation by the chronic pain clinical pharmacist practitioner, instead of during the check-in process. Collecting the UTS at the end of the clinic visit was expected to decrease the time duration for the check-in-process by decreasing the patient work-up requirements for the nurses or the care assistant, so they can be placed into a room sooner. As UTS results take from one to three hours to obtain, these results do not affect the clinical pharmacist practitioner's plan of action decided during the patient visit.

### Measures

After these interventions were implemented into each clinic for two weeks, a follow-up PFA was performed for a one week period. Data for the mean duration time for each phase, and mean visit time were compared with baseline data. Intervention-based improvements in the efficiency of patient flow were associated with decreases in mean duration time for specific phases and mean visit time. To determine the statistical significance of the implemented interventions on patient flow, the baseline and follow-up data for mean time of the patient visit were compared using a two-sample t-test with equal variances.

## Results

### Anticoagulation

**Figure 2:**
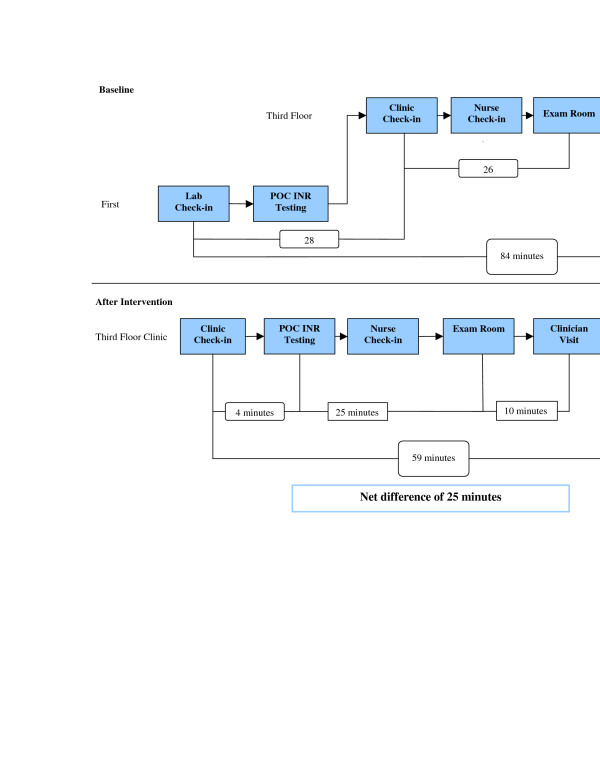
Anticoagulation clinic patient flow.

At baseline, the mean visit time for 93 anticoagulation clinic patient visits was 84 minutes (+/- 50 minutes). Of the total visit, 62 minutes (76% of the visit time) occurred before being evaluated by an anticoagulation clinical pharmacist practitioner. The majority of the wait time occurred between the laboratory check-in and the anticoagulation clinic check-in (28 min) and between the anticoagulation clinic check-in and being placed into an examination room (26 min). After concurrent implementation of both interventions, follow-up for 96 clinic patient visits found the mean visit time was 59 minutes (+/-25 min). The mean visit time decreased by 25 minutes (p < 0.001). The amount of time before being evaluated by an anticoagulation clinical pharmacist practitioner was reduced to 39 minutes (66% of total visit time). The most significant improvement was made in decreasing the wait time between check-in and POC INR testing, from 28 to 4 minutes. Follow-up data also found wait time for placement into an examination room from anticoagulation clinic check-in increased from 26 to 29 minutes. Time usage changes in clinic patient flow after implementation of interventions is outlined by Figure 2.

### Chronic pain

**Figure 3:**
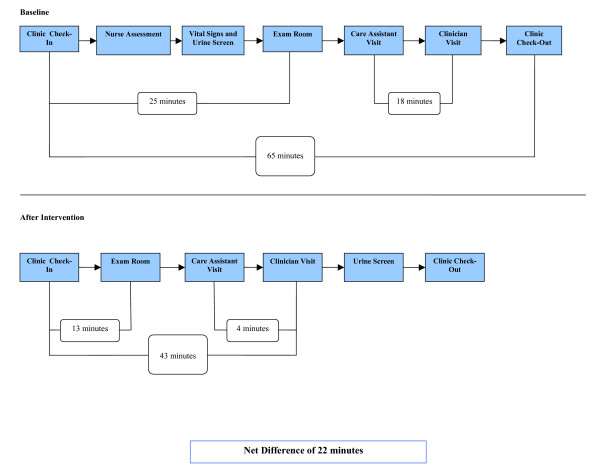
Chronic pain management clinic patient flow.

At baseline, the mean visit time for 25 chronic pain clinic patient visits was 65 minutes (+/- 21 min), for established patient visits and patients arriving no earlier than 45 minutes for their appointment. Patients arriving earlier than 45 minutes were excluded from analysis because their wait time for placement into an examination room after check-in was artificially inflated. Of the total visit, 48 minutes (74% of the visit time) took place before evaluation by a clinical pharmacist practitioner. The majority of the wait time occurred between chronic pain clinic check-in and being placed into a room (25 min) and between being seen by the care assistant and the clinical pharmacist practitioner (18 min). After concurrent implementation of both the interventions, follow-up of 19 chronic pain clinic patient visits meeting inclusion criteria found the mean visit time of the patient visit was 43 minutes (+/- 14 min). The mean visit time decreased by 22 minutes (p < 0.001). The amount of time before being evaluated by a chronic pain clinical pharmacist practitioner was reduced to 27 minutes (63% of visit time). The most significant improvements were made in decreasing the wait time between pain clinic check-in and placement into a room, from 25 to 13 minutes, and decreasing time between the care assistant and clinician assessment, from 18 to 4 minutes. Time usage changes in clinic patient flow after implementation of interventions is outlined by Figure 3.

## Discussion

Use of PFA was an effective technique to identify areas within the care process for improvement, target interventions, and measure effectiveness of interventions. It allowed for a decrease in patient wait time to receive care. Studies can be completed within the context of usual practice without substantial requirements for additional personnel or costs, and produce useful data in a short period of time. The ease of performing PFA in clinic negates the need to hire external consultants to identify areas of inefficiency in the process of patient care, allowing clinic staff familiar with the patient flow process to develop a sense of ownership to resolve these problems. As such, they offer a widely usable technique for improving office practice.

Study limitations include use of a small sample size and collecting data from only one clinic involving a primary care-based disease management program. This analysis was only performed once to assess and improve the patient efficiency in clinic because a significant improvement in visit efficiency occurred after the initial interventions. Optimally, PFA should be administered on an interval basis to ensure the sustainability of patient flow efficiency that was achieved from the interventions. Patient and staff satisfaction with the intervention was not assessed. Changes in patient throughput were not formally assessed and are confounded by the addition of more mid-level providers shortly after performing PFA. Future studies will focus on the formal assessment of the costs of interventions and changes in revenue generated by clinic to see if improvements in patient visit efficiency help increase overall clinic revenue.

## Competing interests

The author(s) declare that they have no competing interests.

## Authors' contributions

NMP developed study design, administered surveys, performed data management, assisted in drafting, editing, and revision of the manuscript. RMM developed study design, supervised conduction of study, performed data management, and edited the manuscript. BB developed study design, administered surveys, assisted in drafting, editing, and revision of manuscript. TJI developed study design and assisted in drafting, editing, and revision of manuscript. PRC performed statistical analyses and assisted in revising and editing manuscript. DAD assisted in interpreting the data and revising and editing manuscript. MPP developed study design, supervised conduction of study, assisted in drafting, revising, and editing manuscript.

## Pre-publication history

The pre-publication history for this paper can be accessed here:


